# The risk of second primary malignancies in colorectal cancer patients using calcium channel blockers

**DOI:** 10.1038/s41598-023-29535-7

**Published:** 2023-03-01

**Authors:** Jana Halámková, Lucia Bohovicová, Lucie Pehalová, Tomáš Kazda, Roman Goněc, Teodor Staněk, Lucie Mouková, Dagmar Adámková Krákorová, Šárka Kozáková, Marek Svoboda, Regina Demlová, Lucie Gabrielová, Lenka Hernychová, Igor Kiss

**Affiliations:** 1 Department of Comprehensive Cancer Care, Masaryk Memorial Cancer Institute, Brno, Czech Republic; 2 Department of Comprehensive Cancer Care, Faculty of Medicine, Masaryk University, Brno, Czech Republic; 3 Institute of Health Information and Statistics of the Czech Republic, Prague, Czech Republic; 4 Institute of Biostatistics and Analyses, Faculty of Medicine, Masaryk University, Brno, Czech Republic; 5 Department of Radiation Oncology, Masaryk Memorial Cancer Institute, Brno, Czech Republic; 6 Department of Radiation Oncology, Faculty of Medicine, Masaryk University, Brno, Czech Republic; 7 Department of Pharmacy, Masaryk Memorial Cancer Institute, Brno, Czech Republic; 8 Department of General Surgical Oncology, Masaryk Memorial Cancer Institute, Brno, Czech Republic; 9 Department of Surgical Oncology, Faculty of Medicine, Masaryk University, Brno, Czech Republic; 10 Department of Gynecologic Oncology, Masaryk Memorial Cancer Institute, Brno, Czech Republic; 11 Department of Pharmacy, University Hospital Brno, Brno, Czech Republic; 12 Department of Pharmacology, Faculty of Medicine, Masaryk University, Brno, Czech Republic; 13 Clinical Trial Unit, Masaryk Memorial Cancer Institute, Brno, Czech Republic; 14 Department of Breast, Skin and Oncoplastic Surgery, Masaryk Memorial Cancer Institute, Brno, Czech Republic; 15 Research Centre for Applied Molecular Oncology, Masaryk Memorial Cancer Institute, Brno, Czech Republic; 16 Masaryk Memorial Cancer Institute, Žlutý Kopec 7, 656 53 Brno, Czech Republic

**Keywords:** Cancer, Cancer epidemiology, Cancer screening, Gastrointestinal cancer, Risk factors, Oncogenesis

## Abstract

Calcium channel blockers are among the most commonly used agents in the treatment of cardiovascular diseases. There are several known side-effects associated with their long-term use, whereas other potential adverse effects are yet to be proven. This study aims to evaluate the association between calcium channel blockers exposure and the incidence of second primary malignancy. We established a cohort of 1401 patients with colorectal cancer diagnosed in our institution between January 2003 and December 2016. Patients were followed-up until December 2020. The tumor characteristics and basic clinical data including medication information were obtained from the hospital information system database. Second malignancy was detected in 301 patients (21.5%), and occurred in 27.8% of patients who used calcium channel blockers compared to only 19.9% among non-users. Their use was associated with an increased incidence of bladder cancer in particular. Subanalysis of patients with second malignancy displayed a higher proportion of right-sided colon cancer compared to rectal carcinoma in non-users. Survival analysis revealed significantly better outcomes in early-stage colorectal cancer patients without a history of calcium channel blockers treatment or second primary malignancy.

## Introduction

Colorectal cancer (CRC) belongs to the most common cancer diagnosis in the Czech Republic (10.7 million inhabitants), with the absolute incidence reaching 6970 cases in 2020^1^. From the international perspective, Czech colorectal cancer age-standardized incidence rate (ASR) in 2020 remained considerably above the global average (33.7 in Czechia vs. 19.5 globally), ranking the Czech republic 14th in Europe and 17th globally^2^. The effectiveness of screening programs and personalized therapy have reduced CRC mortality and significantly improved the survival of cancer patients, thus increasing their risk of developing a second primary malignancy (SPM). Data on the type and frequency of SPMs and potential risk factors for their development are essential for high-quality survivorship care and patient-tailored posttreatment cancer surveillance. The risk of a new primary cancer in patients with a previously diagnosed carcinoma is about 20%, and approximately 30% of cancer survivors aged > 60 years experience more than one other cancer diagnosis in their remaining lifetime^3^.

Calcium is an intracellular ion and second messenger that influences a variety of cellular functions. Calcium signaling and the modulation of intracellular calcium levels are essential in the processes of carcinogenesis, and may contribute to the development of drug resistance^4^. Calcium channel blockers (CCBs) are widely prescribed drugs, used predominantly in the treatment of arterial hypertension. The debate on their impact on cancer was ignited in the 1980s^5^ when several authors raised concerns about their carcinogenic potential^6,7^. The hypothesized mechanism revolved around the influence of CCBs on intracellular calcium homeostasis that might interfere with apoptosis^8,9^, or calcium-mediated pathways implicated in tumorigenesis^10–12^. Other possible explanations for the increased risk of cancer among CCB users involve modulation of cytokine production and T-cell immune response induced by CCB^13^, or their impact on the tumor microenvironment^14^. On the other hand, there have been several studies that reported a null association between CCB and overall or specific cancer risk^15–18^. As the relationship between CCB and cancer risk remains controversial, we conducted a retrospective analysis of a large cohort of CRC patients to evaluate the impact of CCBs on secondary malignancy in patients with primary CRC.

## Material and methods

### Study population

We identified a population of adult patients with histologically confirmed CRC who were treated at the Masaryk Memorial Cancer Institute (MMCI), Czech Republic, between January 2003 and December 2016. All cohort members have signed the informed consent to participate in the research project. The study was conducted in accordance with the Declaration of Helsinki and approved by the Ethics Committee of Masaryk Memorial Cancer Institute (2019/1827/MOU, date of approval: 18th Jun 2019). Patients with CRC diagnosed at autopsy, lost to follow-up, and with a high risk of the development of SPMs due to hereditary cancer syndrome (e.g. BRCA1, 2, Lynch syndrome, or familial adenomatous polyposis) were excluded from the study. The patients were followed from the time of primary cancer diagnosis until December 2020. We used the hospital electronic health records to identify patients’ medication history and basic parametric health data. Exposure to CCB had to precede the index date and we confined our analysis to patients using dihydropyridines.

### Definition of second primary malignancy

We followed the SEER multiple primary and histology coding rules^19^, i.e. only tumors with (1) ICD-O-3 histology codes that differ in the first, second, or third number and (2) tumors with ICD-O-3 topography codes that differ in the second and/or third characters were considered multiple primaries^20^. Primary cancer metastases, corresponding to ICD codes C79.0–C79.9 (secondary malignant neoplasms) were excluded from the analysis. All lesions evaluated as second primary malignancies were histologically verified.

### Statistical analysis

Comparisons of population characteristics categorized by the use of SPM were summarized with counts and frequencies and tested with the Fisher exact test. Continuous characteristics were analyzed using the Mann–Whitney test, median, and 25–75% percentile. The relationship between SPM and laterality of CRC stratified by the use of CCB was tested with Fisher exact test. Univariate and multivariate logistic regression model was used to quantify the association between SPM and the use of calcium channel blockers. The following covariates were used in the multivariate model: gender, age at CRC diagnosis, clinical stage, relapse status, and laterality. Patients with unknown clinical stage were excluded from the analysis. Patients with the diagnosis of C18.4 (transverse colon) were not included in the analysis of laterality, as the topography did not allow for differentiation between right and left-sided CRC.

Cancer-specific analysis of the occurrence of SPMs and the use of CCBs was performed by the N-1 chi-squared test. SPMs with an unknown date of diagnosis were excluded (7 cases). The Czech National Cancer Registry (CNCR)^1^ was used to compare the frequencies of relevant cancers to their prevalence in the general Czech population.

Kaplan–Meier curves were plotted to show the survival of CRC patients with respect to the occurrence of SPM, use of CCBs, and clinical stage. Observations were censored at 15 years of follow-up. The Breslow test was utilized to compare survival data between the subgroups of patients defined by the use of CCBs and the occurrence of SPM.

## Results

In total, 1401 patients were identified and enrolled in this study. The cohort involved 855 men (61%) and 546 women; the median age was 64 years. Basic patient characteristics according to the occurrence of SPM are summarized in Table 1. Treatment with CCB was reported in 277 patients (19.8%). The subgroups of patients stratified by the use of CCB were well balanced in terms of gender (60.5% of men in CCB-treated group vs. 63.2% of men in non-users, p-value 0.449), the median age was significantly lower in the subgroup of non-users (63 (55–71) vs. 67 (60–74.5), p < 0.001). SPMs were diagnosed in 301 patients (21.5%). The median follow-up was 9.01 years. During the study period, 723 patients died and 73 patients were censored at the 15-year survival endpoint.

**Table 1:** Characteristics of colorectal cancer patients (C18–C20) stratified by occurrence of second primary malignancy.

	No SPM (N = 1100)	With SPM (N = 301)	p-value
Gender			
Men	680 (61.8%)	175 (58.1%)	0.257^1^
Women	420 (38.2%)	126 (41.9%)	
Age at CRC diagnosis			
18–44	90 (8.2%)	16 (5.3%)	** < 0.001**^1^
45–54	165 (15.0%)	25 (8.3%)	
55–64	350 (31.8%)	67 (22.3%)	
65–74	323 (29.4%)	125 (41.5%)	
75 +	172 (15.6%)	68 (22.6%)	
Median (25–75% percentile)	63 (55–71)	69 (61–74)	** < 0.001**^2^
Clinical stage			
Complete records	1066 (96.9%)	287 (95.3%)	**0.014**^1^
Stage I + in situ	273 (25.6%)	75 (26.1%)	
Stage II	266 (25.0%)	89 (31.0%)	
Stage III	304 (28.5%)	85 (29.6%)	
Stage IV	223 (20.9%)	38 (13.2%)	
Not available	34 (3.1%)	14 (4.7%)	
Grade			
Complete records	762 (69.3%)	241 (80.1%)	0.121^1^
1	190 (24.9%)	52 (21.6%)	
2	424 (55.6%)	152 (63.1%)	
3	148 (19.4%)	37 (15.4%)	
Not available	338 (30.7%)	60 (19.9%)	
Relapse			
Yes	362 (32.9%)	64 (21.3%)	** < 0.001**^1^
No	738 (67.1%)	237 (78.7%)	
Use of calcium channel blockers			
Yes	200 (18.2%)	77 (25.6%)	**0.005 **^**1**^
No	900 (81.8%)	224 (74.4%)	

*SPM* second primary malignancy, *CRC* colorectal cancer.

^1^Fischer exact test, ^2^Mann-Whitney test.

Significant values are bold.

A single secondary neoplasm was found in 246 (17.6%) cases, 47 (3.4%) patients suffered from two SPMs, and 8 (0.6%) presented with three SPMs. A significantly higher incidence of SPMs was observed among the elderly and in patients with a history of early-stage CRC.

SPMs occurred in 77 patients who used a calcium channel blocker (27.8%), compared to only 224 among 1124 non-users (19.9%) (p = 0.005). Univariate logistic regression models showed a significantly higher incidence of SPMs in patients treated with CCBs compared with non-users (p = 0.017). Multivariate regression analysis confirmed the positive association between CCB and SPM, however, the results did not reach statistical significance, with odds ratio for the occurrence of SPM being 1.32 (95% CI   0.96–1.82, p = 0.091) for patients using CCB.

As demonstrated in Table 2, a statistically significant relationship between the occurrence of SPM and the laterality of CRC was detected in patients who were not treated with CCB. These patients who suffered from SPM had a higher proportion of right colon (25.7% vs. 17.9%) and left colon cancer (32.9% vs. 29.2%) and a lower proportion of rectal cancer (41.4% vs. 52.9%) compared to patients without SPM. This association was statistically significant (p = 0.006).An opposite trend was observed in patients treated with CCB, in whom SPM incidence was mainly associated with rectal cancer (51.4% vs. 47.1%), although the relationship did not reach statistical significance (p = 0.802).

**Table 2:** The relationship between second primary malignancies and laterality of colorectal cancer stratified by the use of calcium channel blockers excluding patients with C18.4 (transverse colon).

	No use of calcium channel blockers (N = 1074)			Use of calcium channel blockers (N = 265)			Total (N = 1339)		
	No SPM (N = 864)	With SPM (N = 210)	p-value*	No SPM (N = 191)	With SPM (N = 74)	p-value*	No SPM (N = 1055)	With SPM (N = 284)	p-value*
Right colon	155 (17.9%)	54 (25.7%)	**0.006**	39 (20.4%)	13 (17.6%)	0.802	194 (18.4%)	67 (23.6%)	**0.040**
Left colon	252 (29.2%)	69 (32.9%)		62 (32.5%)	23 (31.1%)		314 (29.8%)	92 (32.4%)	
Rectum	457 (52.9%)	87 (41.4%)		90 (47.1%)	38 (51.4%)		547 (51.8%)	125 (44.0%)	

*SPM* second primary malignancy.

*Statistical significance for the colorectal cancer site distribution was evaluated using Fisher exact test.

Significant values are bold.

**Figure 1:**
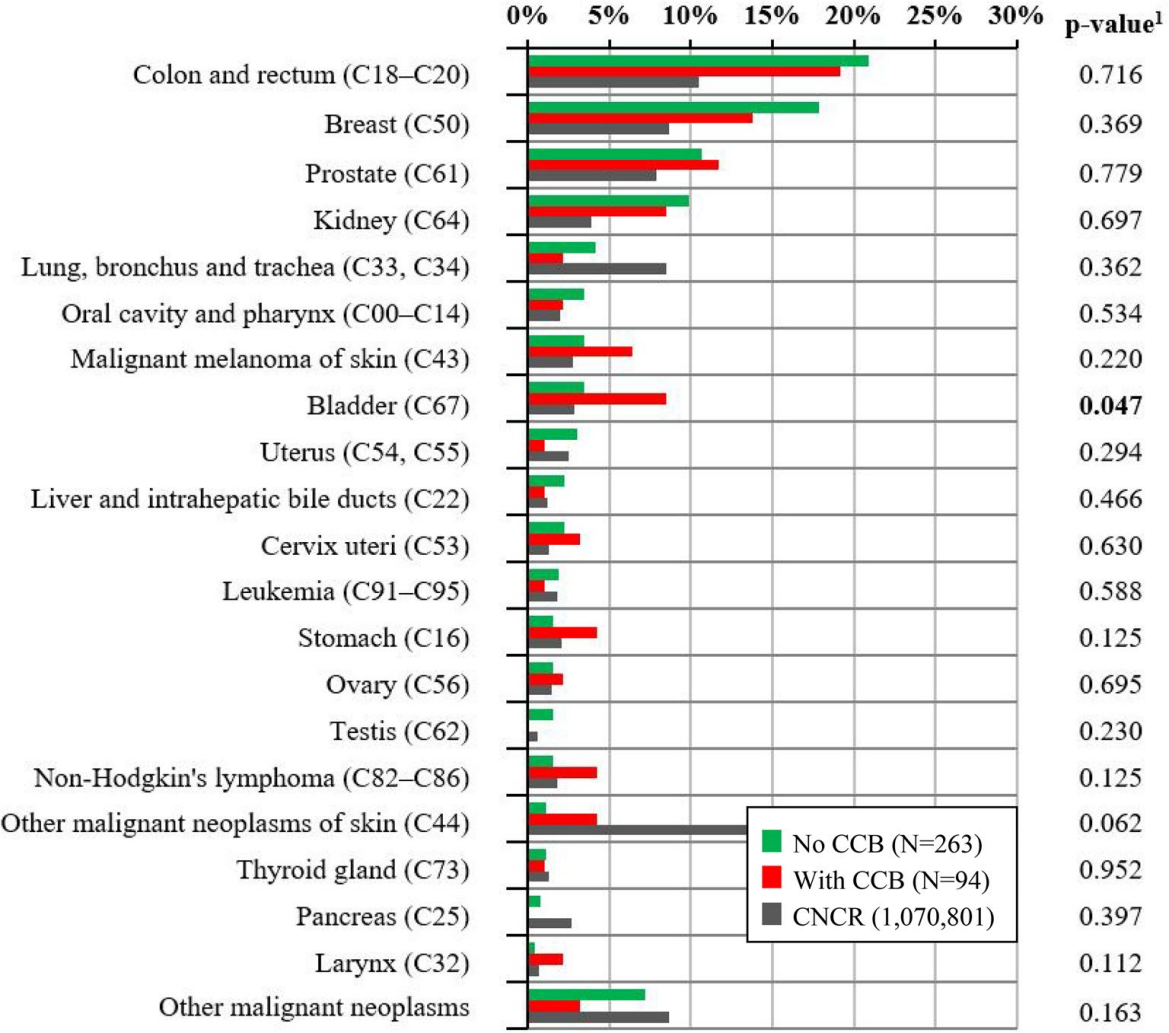
Occurrence of second primary malignancies with respect to use of calcium channel blockers. Only SPMs with a known date of diagnosis were considered (the date of diagnosis was not available for 7 SPMs). *SPMs* second primary malignancies, *CCB* calcium channel blocker, *CNCR* Czech National Cancer Registry (2003–2016). ^1^p-value of N-1 Chi-squared test for group no calcium channel blockers and group with calcium channel blockers.

The prevalence of site-specific second cancers in CCB users and non-users is summarized in Table 3, whereas the last column (CNCR) serves as a reference, indicating the frequency of particular neoplasia in the general Czech population throughout the corresponding period. A bar chart for visual presentation of the data, stratified by the use of CCB is provided in Fig. 1. The most common second malignancy was CRC in both groups of patients. A statistically significant increase in the incidence of SPM among CCB users was detected in the subgroup of patients with bladder cancer. The analysis of bladder cancer incidence in relation to the use of CCB separately for men and women revealed that a positive correlation was significant only in women (0.9% vs. 10.3% in CCB users, p = 0.004), whereas in men the incidence of bladder cancer was not related to the use of CCB (5.4% vs. 7.3%, p = 0.625).

**Table 3:** Second primary malignancies by the site of diagnosis stratified by the use of calcium channel blockers.

	No use of calcium channel blockers (N = 263)	Use of calcium channel blockers (N = 94)	All malignant cancers according to CNCR (N = 1,070,801)
Oral cavity and pharynx (C00–C14)	9 (3.4%)	2 (2.1%)	20,962 (2.0%)
Stomach (C16)	4 (1.5%)	4 (4.3%)	22,385 (2.1%)
Colon and rectum (C18–C20)	55 (20.9%)	18 (19.1%)	112,410 (10.5%)
Liver and intrahepatic bile ducts (C22)	6 (2.3%)	1 (1.1%)	12,500 (1.2%)
Pancreas (C25)	2 (0.8%)	0 (0.0%)	28,463 (2.7%)
Larynx (C32)	1 (0.4%)	2 (2.1%)	7539 (0.7%)
Lung, bronchus and trachea (C33, C34)	11 (4.2%)	2 (2.1%)	91,145 (8.5%)
Malignant melanoma of skin (C43)	9 (3.4%)	6 (6.4%)	29,507 (2.8%)
Other malignant neoplasms of skin (C44)	3 (1.1%)	4 (4.3%)	289,780 (27.1%)
Breast (C50)	47 (17.9%)	13 (13.8%)	92,356 (8.6%)
Cervix uteri (C53)	6 (2.3%)	3 (3.2%)	13,585 (1.3%)
Uterus (C54, C55)	8 (3.0%)	1 (1.1%)	26,677 (2.5%)
Ovary (C56)	4 (1.5%)	2 (2.1%)	15,482 (1.4%)
Prostate (C61)	28 (10.6%)	11 (11.7%)	84,720 (7.9%)
Testis (C62)	4 (1.5%)	0 (0.0%)	6,614 (0.6%)
Kidney (C64)	26 (9.9%)	8 (8.5%)	41,511 (3.9%)
Bladder (C67)	9 (3.4%)	8 (8.5%)	30,948 (2.9%)
Thyroid gland (C73)	3 (1.1%)	1 (1.1%)	13,379 (1.2%)
Non-Hodgkin’s lymphoma (C82–C86)	4 (1.5%)	4 (4.3%)	19,011 (1.8%)
Leukemia (C91–C95)	5 (1.9%)	1 (1.1%)	19,041 (1.8%)
Other malignant neoplasms	19 (7.2%)	3 (3.2%)	92,786 (8.7%)

Only SPMs with a known date of diagnosis were considered (the date of diagnosis was not available for 7 SPMs).

*SPM* second primary malignancy, *CNCR* Czech National Cancer Registry (2003–2016).

**Figure 2:**
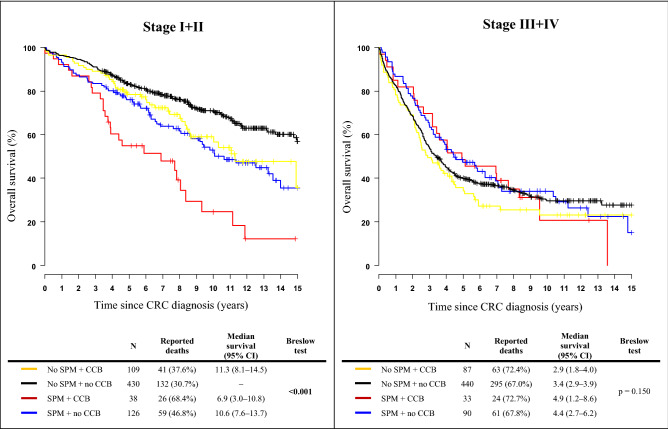
Kaplan–Meier curves of 15-year survival among colorectal cancer patients (C18–C20) stratified by the occurrence of second primary malignancy and the use of calcium channel blockers according to clinical stages. *SPM* second primary malignancy, *CCB* calcium channel blocker, *CI* confidence interval.

The association between overall survival and the use of CCB with respect to SPM occurrence is shown in Fig. 2. Patients were stratified according to clinical stage of the CRC. Overall survival was significantly better in the subgroup of CRC patients with early-stage carcinoma, without SPMs, and those without CCB therapy. On the contrary, the worst survival was seen in patients with SPMs using calcium channel blockers, however, statistical significance was restricted to patients with early-stage disease.

## Discussion

In this large single-institutional retrospective cohort study, we found a positive association between the incidence of second cancer and the use of CCB in CRC patients. The observed correlation supports the hypothesis of a tumor-promoting potential of these drugs indicated by the meta-analysis of Rotshild et al. in lung cancer^21^, and in a large population-based case–control study by Li et al. who reported an increased risk of breast cancer in long-term users of CCB^22^. Pooled data from twenty studies on CCB use and breast cancer risk also indicated a positive association^23^. Similarly, a meta-analysis by Yang et al.^24^ suggested a 13% increase in the risk of prostate cancer among patients treated with CCB for more than 5 years.

Our subgroup analysis showed a statistically significant increase in bladder cancer in particular among CCB users. Interestingly, the association was significantly pronounced in female patients, who are generally less susceptible to this type of malignancy. This might support the notion that there are other strong molecular and epidemiologic factors underlying gender disparities in bladder cancer incidence in favor of men. On the other hand, these findings are in contrast with the work of Guercio et al.^25^, who indicated a chemoprotective effect of CCB in relation to bladder cancer, even after adjusting for several established risk factors shared between cardiovascular diseases and cancer. Conversely, the relative risk of renal cancer was higher among CCB users (1.65, 95% CI 1.11–1.66) in the cohort. These results have not been replicated so far, and a meta-analysis of 7 studies did not confirm a statistically significant association between CCB and bladder cancer^26^. Further validation with larger cohort of patients who use calcium channel blockers would help to draw more solid conclusions.

Arguments against the oncogenic potential of antihypertensive drugs also generate the question of whether the association exists irrespective of hypertension. The recent cohort study of Matsui et al.^27^ comprising 140,420 participants showed that the use of antihypertensive drugs increased the hazard ratio for renal cancer, even when adjusted for confounding factors such as blood pressure, smoking status, BMI or history of diabetes. However, the analysis did not discriminate between the types of antihypertensive medication. Another nation-wide cohort study^28^ of 70,549 participants did not demonstrate a significant increase in cancer risk in patients treated for hypertension, even if adjusted for particular drug class and other potential risk factors. Since our preliminary analysis did not confirm a link between SPM and the use of beta-blockers (mature data are yet to be published in a separate report), this could indicate that CCBs represent a specific additional risk factor beyond hypertension itself. These findings are consistent with the results of a comprehensive meta-analysis of studies evaluating the impact of common antihypertensives on cancer risk that reported an association between CCB use and the incidence of prostate and skin cancers when compared to other drug classes^29^. After adjusting our analysis for other independent variables such as gender, age at CRC diagnosis, clinical stage, or relapse status the positive association between CCB treatment and the occurrence of SPM remained, however, the trend lacked statistical power. This might be due to insufficient sample size, as well as confounding by other established risk factors, that were not taken into account in the analysis, e.g. obesity, use of chemotherapy, or smoking status. Yet, the discrepancy in our results might also be interpreted in the context of several other studies that have disputed the relationship between CCBs and cancer in breast^30–34^, prostate^35^, or in malignancies in general^36,37^.

A negative association between the use of CCBs and survival was observed among early-stage CRC patients with SPM, whereas survival in patients with advanced-stage CRC and SPM was not influenced by the use of CCBs. This probably indicates the presence of other significant mortality determinant in patients with multiplicity. The best survival outcomes were achieved in the subgroup of early-stage cancer survivors who did not experience SPM and were not treated with CCB. These findings might also be best interpreted in the context of unrecorded factors and missing data that might have contributed to these results. Besides the healthy-user bias, overall mortality may have been modified by the absence of adjuvant chemotherapy or a lower chance of cancer-associated complications. A subanalysis of cancer-specific mortality might cast new light on the results, unfortunately, this group of subjects is underpowered for comprehensive statistical analysis, leaving it a challenge for further research.

To the best of our knowledge, this is one of very few studies addressing the impact of CCB therapy on the outcomes of CRC patients. There are several limitations to our study. The research was narrowed to a specific population of cancer survivors and the number of patients who used calcium channel blockers was relatively low compared to non-users (277 vs. 1124). Our findings would require a larger and more diverse cohort of patients with CCB medication to draw any general conclusions. Balkrishnan et al.^38^ who evaluated the association between antihypertensive treatment and mortality in a cohort of 13,982 CRC patients reported a protective effect of these drugs; however, patients using CCB were not included in the analysis. Similarly, Peng et al.^39^ reported a beneficial impact of antihypertensives on the prognosis of 713 CRC patients, although their results were not statistically significant.

Despite conflicting results and the above-mentioned limitations, our findings provide specific insight into tertiary cancer prevention that captures one piece of the complex puzzle related to the development of SPM in cancer survivors. The correlation between the use of CCB, overall survival, and the development of SPM might point towards other associated indicators of cancer patient outcomes and contribute to personalized cancer care.

## Conclusion

We showed that CRC patients treated with CCB have a higher incidence of SPM, especially in the elderly and early-stage cancer subgroup. CCB use correlated with the incidence of bladder cancer in particular. Our findings also support the role of regular tertiary care in CRC survivors aimed at screening of SPM, mainly among patients with CCB treatment. Given the widespread use of these antihypertensive agents, further research is warranted to clarify their potential role in oncogenesis and cancer preventive care.

## Data Availability

The datasets generated and analyzed in the current study are available from the corresponding author on reasonable request.
